# Global challenges with scale-up of the integrated management of childhood illness strategy: results of a multi-country survey

**DOI:** 10.1186/1471-2458-11-503

**Published:** 2011-06-27

**Authors:** Ameena E Goga, Lulu M Muhe

**Affiliations:** 1 Health Systems Research Unit, Medical Research Council, 1 Soutpansberg Road, Pretoria, 0001 Pretoria, South Africa; 2 Department of Child and Adolescent Health and Development (CAH), World Health Organisation, Avenue Appia 20, 1211 Geneva 27, Switzerland

## Abstract

**Background:**

The Integrated Management of Childhood Illness Strategy (IMCI), developed by WHO/UNICEF, aims to contribute to reducing childhood morbidity and mortality (MDG4) in resource-limited settings. Since 1996 more than 100 countries have adopted IMCI. IMCI case management training (ICMT) is one of three IMCI components and training is usually residential over 11 consecutive days. Follow-up after ICMT is an essential part of training. We describe the barriers to rapid acceleration of ICMT and review country perspectives on how to address these barriers.

**Methods:**

A multi-country exploratory cross-sectional questionnaire survey of in-service ICMT approaches, using quantitative and qualitative methods, was conducted in 2006-7: 27 countries were purposively selected from all six WHO regions. Data for this paper are from three questionnaires (QA, QB and QC), distributed to selected national focal IMCI persons/programme officers, course directors/facilitators and IMCI trainees respectively. QC only gathered data on experiences with IMCI follow-up.

**Results:**

33 QA, 163 QB and 272 QC were received. The commonest challenges to ICMT scale-up relate to funding (high cost and long duration of the residential ICMT), poor literacy of health workers, differing opinions about the role of IMCI in improving child health, lack of political support, frequent changes in staff or rules at Ministries of Health and lack of skilled facilitators. Countries addressed these challenges in several ways including increased advocacy, developing strategic linkages with other priorities, intensifying pre-service training, re-distribution of funds and shortening course duration. The commonest challenges to *follow-up *after ICMT were lack of funding (93.1% of respondents), inadequate funds for travelling or planning (75.9% and 44.8% respectively), lack of gas for travelling (41.4%), inadequately trained or few supervisors (41.4%) and inadequate job aids for follow-up (27.6%). Countries addressed these by piggy backing IMCI follow-up with routine supervisory visits.

**Conclusions:**

Financial challenges to ICMT scale-up and follow-up after training are common. As IMCI is accepted globally as one of the key strategies to meet MDG4 several steps need to be taken to facilitate rapid acceleration of ICMT, including reviewing core competencies followed by competency-driven shortened training duration or 'on the job' training, 'distance learning' or training using mobile phones. Linkages with other 'better-funded' programmes e.g. HIV or malaria need to be improved. Routine Primary Health Care (PHC) supervision needs to include follow-up after ICMT.

## Background

The Integrated Management of Childhood Illness Strategy (IMCI), developed by WHO and UNICEF, has been identified as a key strategy to meeting the fourth millennium development goal (MDG4). IMCI has three components, viz. case management training (ICMT), strengthening the health system and intensifying household and community behaviours to improve child health [1]. ICMT is presented as an 11-day course (usually residential) and teaches health care providers to manage sick children up to the age of 5 years, presenting to primary health care facilities with illnesses that account for major childhood morbidity and mortality. The course comprises six key modules and clinical practice. WHO recommends that 44.2% of course time is spent on clinical practice 1.2% on Introduction, 20.9% on Assess and Classify, 4.9% on Identify Treatment, 11.6% on Treat the Child, 6.9% on Counsel the Mother, 6.9% on Sick Young Infant and 3.5% on Follow-up. The IMCI management algorithms or charts are colour coded and each trained health care provider is provided with a chart booklet to use during consultations. Each ICMT course is facilitated by trained facilitators and a 1:<4 facilitator: participant ratio is recommended.

Follow-up after training is an essential component of ICMT, as laid down in the IMCI information package [1]. The package describes follow-up after ICMT as an opportunity to reinforce skills acquired during training and solve problems encountered during IMCI implementation. The approach to follow-up developed by the WHO Department of Child and Adolescent Health and Development (CAH), also serves as a bridge to ongoing district-level supervision.

ICMT has been shown to reduce under-five mortality [2] and to improve antimicrobial use in first level facilities [3].

Despite data on the effectiveness of IMCI in decreasing antimicrobial prescription by health workers, improving quality of care, child health indicators, quality of counselling provided to caregivers, and bed net use [3-10], current global coverage by IMCI-case management-trained health workers is low [11]. Furthermore recent data from South Africa showed that although health workers in South Africa were implementing IMCI, clinical assessments using IMCI were frequently incomplete - only 18% checked for all main symptoms [12]. Focus group discussions amongst health workers in South Africa also showed that although they found the training interesting, informative and empowering the training time was short and follow-up visits, though helpful, were often delayed resulting in no ongoing clinical supervision [13].

In view of the potential contribution that IMCI scale-up could have on childhood morbidity and mortality, and the dearth of documented information on how IMCI was actually being implemented globally we conducted a survey in 2006 to review the training approaches and methods used for IMCI case management, document challenges to rapid scale-up of ICMT, document how countries are addressing these barriers and explore country experiences with follow-up after ICMT. It was intended that this information be used to guide future approaches to ICMT.

The first two questions (reviewing training approaches and methods) have been addressed in a separate paper [14]. This paper reports the challenges to rapid ICMT scale-up, how countries have tried to address these, and country experiences with follow-up of IMCI trainees after ICMT.

## Methods

### Study design and aims

The data were obtained from a multi-country exploratory questionnaire survey of in-service IMCI training approaches using qualitative and quantitative methods (Table 1). The survey sought to review country adaptations of IMCI case management training, including adaptations to content and training methodologies (i.e. methods used in addition to the originally proposed written exercises, drills, video, photo booklet, role plays etc.) and to review variations in IMCI training approaches/course duration. The methodology is summarised below. More details are captured in the paper published by Goga et.al.[14]:

**Table 1:** Number Of Questionnaires Received From (% Total Number Of Questionnaire Type), And Length Of Courses Offered, By Region And Country

Region/Country	Questionnaire A n = 33	Questionnaire B n = 163	Questionnaire C n = 272	Total no. forms from country	Adapted duration of ICMT training courses offered
**AFRO**					
Eritrea	2 (6.1)	10 (6.1)	6 (2.2)	18	14 days
Ethiopia	1 (3.0)	13 (8.0)	4 (1.5)	18	6 days
Ghana	1 (3.0)	9 (5.5)	22 (8.2)	32	3 days
Kenya	2 (6.1)	1 (0.6)	1 (0.4)	4	32 hrs
Madagascar	3 (9.1)	7 (4.3)	2 (0.7)	12	3 days, 5-6 days
Niger	0	7 (4.3)	13 (4.8)	20	6 days
Nigeria	1 (3.0)	4 (2.4)	13 (4.8)	18	5-6 days, 14 days
United Republic of Tanzania	1 (3.0)	15 (9.2)	31 (11.5)	47	
Uganda	1 (3.0)	3 (1.8)	2 (0.7)	6	6 days, 14 days
Zambia	0	4 (2.45)	6 (2.2)	10	5-7 days
**WPRO**					
Cambodia	2 (6.1)	5 (3.1)	16 (6.0)	23	
China	1 (3.0)	18 (11.0)	15 (5.6)	34	5-7 days
Fiji	1 (3.0)	2 (1.2)	1 (0.4)	4	5 days
Papua New Guinea	0	0	5 (1.9)	5	5-7 days
Vietnam	1 (3.0)	24 (14.7)	71 (26.4)	96	
**SEARO**					
India	2 (6.1)	1 (0.6)	8 (3.0)	11	6-8 days
Indonesia	1 (3.0)	2 (1.2)	1 (0.4)	4	3 days, 5-6 days
Nepal	1 (3.0)	0	0	1	7 days
**EURO**					
Kazakhstan	2 (6.1)	18 (11.0)	8 (3.0)	28	5-6 days
Kosovo	2 (6.1)	3 (1.8)	2 (0.7)	7	8-10 days
Republic of Moldova	2 (6.1)	9 (5.5)	13 (4.8)	24	12 days
Uzbekistan	3 (9.1)	5 (3.1)	29 (9.7)	37	4 days
**EMRO**					
Sudan	1 (3.0)	0	0	1	5 days
Jordan	0	0	0		5-7 days
Egypt	0	0	0		5-7 days
**AMRO/PAHO**					
Peru	1 (3.1)	2 (1.2)	0	3	
Nicaragua	1 (3.1)	1 (0.6)	3 (1.2)	5	3-5 days

### Countries

All six WHO regions were included so that a global picture of IMCI training approaches could be ascertained. WHO(CAH) purposively selected 27 countries (including one sub-national region - Kosovo). Country selection criteria included a high under-five mortality rate and presence of a WHO National Professional Officer (NPO).

### Study population

The study population comprised purposively selected key informants from each purposively selected country. Key informants were the National focal person for IMCI (one per country) and/or WHO National Programme Officer for IMCI (one per country); IMCI course directors or facilitator (two per region/province within each country and IMCI-trained health workers. The course directors/facilitators should have ever directed/facilitated two or more IMCI case management courses.

### Study procedures

Data were collected over 5-months (January-May 2007). Data for this analysis was obtained from Questionnaire A (QA) for the National IMCI focal person/NPO, Questionnaire B (QB) for course directors/facilitators and Questionnaire C (QC) for IMCI-trained health workers. All forms (questionnaires) gathered quantitative data, using tick boxes and closed-ended questions, and qualitative data using open-ended questions and projective techniques to gather information on current implementation of ICMT and attitudes towards current training approaches.

### Data analysis

Data were entered using EpiData v3.1. Data from QA, QB and QC were captured separately. Data from QA were analyzed using EpiData Analysis v1.1 (Build 68). In addition each QA was scrutinised to glean important details about the nationally recognised courses offered. Data from QB and QC were imported into SAS version 9.1 (SAS Institute Inc., Cary NC, USA) for data management and analysis. Every 10^th ^QC was selected to examine responses to pictures and words about IMCI.

## Results

Table 1 shows the respondents by WHO region and country, and the change in duration of ICMT courses that each country is implementing. The course adaptations are discussed in a previous paper [14].

IMCI national focal persons (usually Ministry of Health staff), WHO National Professional Officers (NPOs), course directors and facilitators mostly thought that IMCI training is not progressing as quickly and smoothly as planned.

### Perceived challenges to rapid scale-up of IMCI implementation

Tables 2 and 3 show the challenges to IMCI implementation, as perceived by IMCI national focal persons/WHO NPOs, in general (Table 2) and by country (Table 3). Inadequate funding for training was perceived as a challenge to rapid scale-up of IMCI implementation by 71% of National Ministry of Health (MOH)/NPO staff and course directors/facilitators. The commonest challenges to rapid ICMT scale-up, as perceived by IMCI national focal persons/NPOs, were the high cost of the course, inadequate funds for training and the long duration of the course (Tables 2 and 3).

**Table 2:** Perceived Challences To IMCI Implementation Globally

	Opinion of national Ministry of Health/WHO National Professional Officer	Opinion of Course Directors/Facilitators
**POLITICAL**		
Lack of buy-in from national stakeholders	14 (45.2)	38 (23.5)
Competing priorities	7 (22.6)	45 (27.8)
**COST**		
Inadequate funds for training	22 (71.0)	115 (71.4)
Too expensive	23 (74.2)	55 (34)
Inadequate fund for printing modules	12 (38.7)	62 (38.3)
Inadequate funds for refreshments	11 (35.5)	52 (32.1)
Inadequate fund for copying video	10 (32.3)	45 (27.8)
Prohibitive financial regulations	13 (41.9)	43 (26.5)
**HR**		
Lack of facilitators	15 (48.4)	16 (9.9)
Lack of clinical instructors	13 (41.9)	30 (18.5)
Demands too high caliber trainers	11(35.5)	38 (23.5)
**OTHER**		
Inadequate facilities for copying the training video	8 (25.8)	28 (17.3)
Long duration of course	16 (51.6)	40 (24.7)
Lack of clinical materials.	5 (16.1)	30 (18.5)
Lack of transport	6 (19.4)	22 (13.6)

**Table 3:** Perceived Challenges To Rapid Scale-Up Of IMCI Implementation By Region And Country - Data From MoH/NPO

							Inadequate Funds for training, printing, refreshments, accommodation, video					**Lack of material, transport, clinical instructors:**				
			
REGION	COUNTRY	Lack of buy-in from national stakeholders	Competing priorities	Long duration of course	Prohibitive financial regulations	Too expensive	Training	Printing Modules	Refreshments	Accommodation	Copying Video	Clinical materials	Transport to clinics	Trainers	CI	Course demands highly skilled trainers
			
AFRO	Eritrea		√	√	√		√	√	√				√	√		
	
	Ethiopia		√	√	√	√	√	√			√		√	√	√	√
	
	Ghana					√	√								√	√
	
	Kenya^a^	√		√		√	√		√	√		√			√	√
	
	Madagascar	√		√	√	√	√		√	√				√	√	√
	
	Niger		√	√	√	√	√									
	
	Nigeria	√		√	√	√	√	√					√	√		√
	
	United Rep. of Tanzania	√				√	√				√			√		√
	
	Uganda	√					√	√	√		√					√
	
	Zambia															
WPRO	Cambodia	√	√	√	√	√	√					√		√	√	√
	
	China			√		√	√	√	√	√	√					
	
	Fiji		√	√			√							√	√	
	
	Papua New Guinea															
	
	Vietnam	√		√		√										

SEARO	India			√	√					√			√	√	√	
	
	Indonesia	√		√	√	√	√	√	√	√	√	√	√	√	√	√
	
	Nepal				√	√										

EURO	Kazakhstan						√	√	√		√			√		
	
	Kosovo	√		√		√	√	√								
	
	Republic of Moldova	√				√	√	√	√	√				√		
	
	Uzbekistan	√				√	√	√		√	√	√				

EMRO	Sudan		√	√	√	√	√		√	√	√	√		√		√

PAHO	Peru		√				√							√		
	
	Nicaragua						√							√		

Note: Trainers = Facilitators; CI = Clinical Instructor

**Figure 1:**
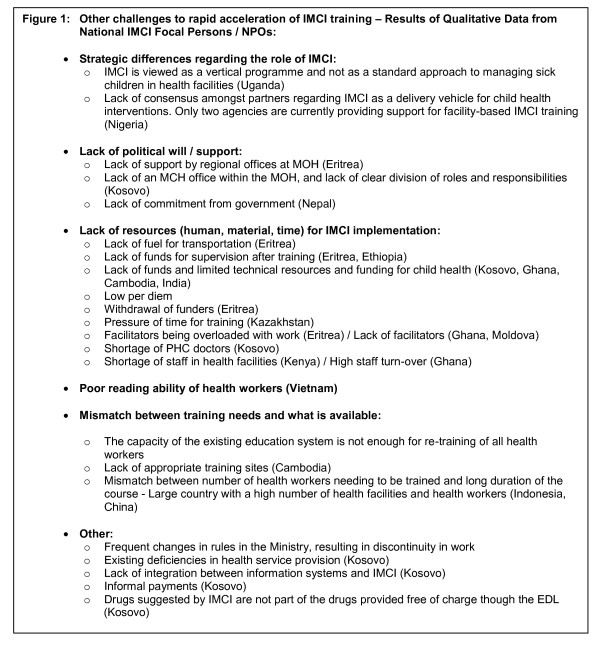
**Other challenges to rapid acceleration of IMCI training - Results from Qualitative Data from National Professional Officers/Ministries of Health**.

The commonest barrier to scale-up, as perceived by course directors and facilitators was funding - specifically inadequate funds for/high cost of training. Qualitative work (open ended questions and projective techniques) highlighted five themes that emerged as barriers to rapid acceleration of IMCI case management training. These included strategic differences regarding the role of IMCI (vertical programme versus integrative approach), lack of political support, lack of human and material resources and time for IMCI implementation, poor reading ability of health workers (mainly in one country.. although other countries had addressed this by shortening the course and decreasing the reading) and mismatch between training needs and resources available (Figure 1).

### How challenges to ICMT scale-up have been addressed

**Figure 2:**
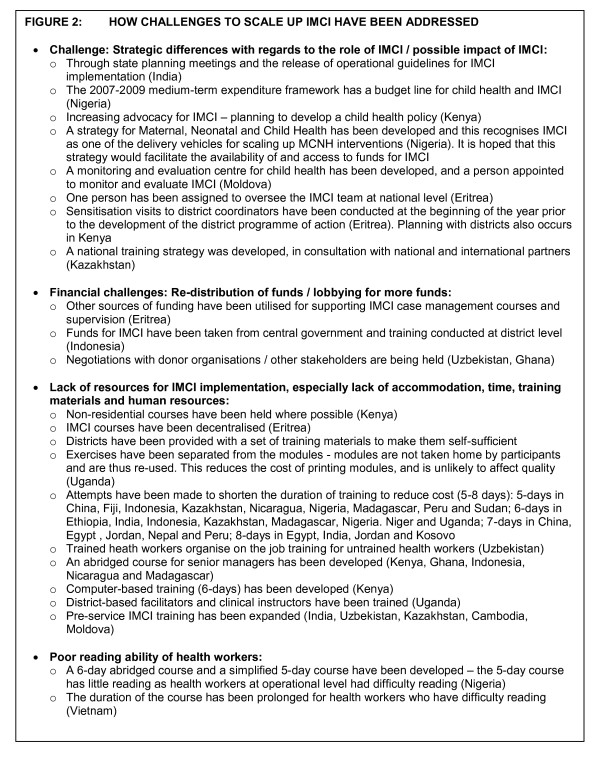
**How challenges to scale up IMCI have been addressed**.

IMCI national focal persons and NPOs reported that challenges have been addressed in several ways, including increased advocacy for IMCI or creation of structural/administrative links between district or national functioning and IMCI, redistribution of funds or increased donor support, non-residential courses, shortening the duration of training and reducing the amount of reading during training (Figure 2).

### Challenges to Follow-up after Training

According to respondents 48.7% (95% CI 35.9-61.7%) of IMCI trainees are followed up within 6 weeks of IMCI case management training, and only 52.5% (IQR 44%) of follow-up visits was linked with routine supervision. IMCI national focal persons/NPOs reported that the commonest challenges to IMCI follow-up after training were lack of funding for follow-up (93.1% of respondents), inadequate funds for travel (75.9%), inadequate funds for planning (44.8%), lack of gas for travel (41.4%), inadequately trained supervisors (41.4%), an inadequate number of skilled supervisors (41.4%) and inadequate job aids for follow-up (27.6%).

**Figure 3:**
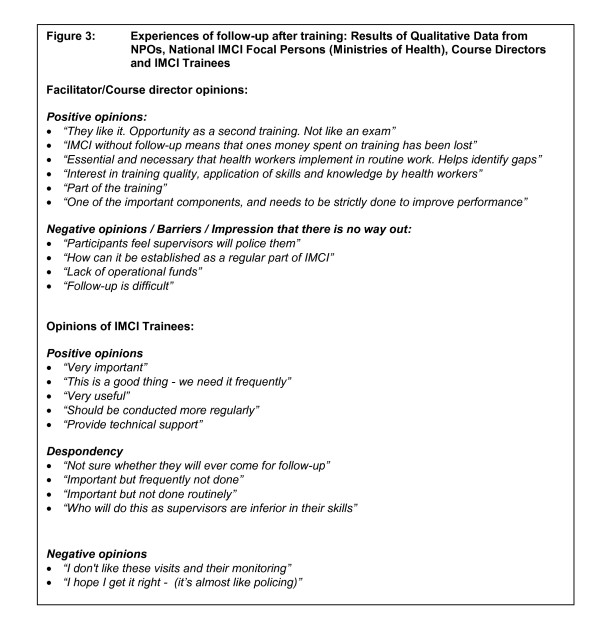
**Experiences of follow-up after training: Results of Qualitative Data from National Professional Officers, Ministries of Health, Course Directors and IMCI Trainees**.

**Figure 4:**
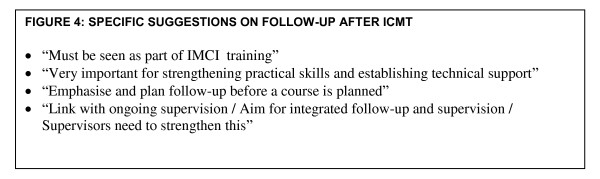
**Specific suggestions on follow-up**.

IMCI national focal persons and NPOs reported several attitudes and experiences of follow-up after training. Whilst some facilitators and course directors accepted that follow-up is an integral part of training other facilitators and course directors seemed unable to plan for integration of IMCI-follow-up activities into their daily work (Figure 3). Similarly although IMCI trained health workers recognized the importance of follow-up a group of trained health workers also expressed despondency about follow-up, likening it to policing. They also highlighted the lack of skill or possible lack of human resources to undertake follow-up (Figure 3). Furthermore four main suggestions about follow-up after ICMT emerged, including viewing follow-up as an important part of IMCI training to strengthen practical skills and establish technical support, planning follow-up before a course is planned and linking follow-up with ongoing routine supervision of services (Figure 4).

## Discussion

Countries have identified several challenges to rapid scale-up of ICMT. The most concerning of these, and one that can possibly be addressed is the high cost of the course. Our previous paper highlighted the acceptability of standardised shortened IMCI courses that include participatory methodologies and adequate clinical practice, amongst selected country respondents [14]. However the effectiveness of shortened courses are still under further investigation. Although a randomized trial in Zambia - which compared performance of primary health workers trained in the 11-day course with those trained in the six-day abridged course - found no significant difference in 10 of 12 priority and 14 of 15 supplemental indicators assessing health worker performance [15], and although research in Kosovo found that assessment was either performed equally well or better by primary health care physicians trained in the 8-day course compared with those trained in the 11-day course [16], a systematic review of the effectiveness of shortening Integrated Management of Childhood Illness guidelines training found that the standard in-service IMCI training course is somewhat more effective than short training; although the magnitude of the difference was unclear (-3 to +18%-points) [17]. Thus shortening duration of training as a means to reduce cost and facilitate scale-up of IMCI still needs to be further investigated.

Health Systems constraints, such as transport or supervision, to scale-up and follow-up after training have been described in other studies: Bryce et.al found that four of the five IMCI multi-country evaluation countries (the exception is Tanzania) had difficulties in expanding the strategy at national level while maintaining adequate intervention quality [18]. They found that the full weight of health system limitations on IMCI implementation was not appreciated at the outset, and only after investigation was it clear that finding solutions to larger problems such as political commitment, human resources, financing, integrated or at least coordinated programme management, and effective decentralization are essential underpinnings of successful efforts to reduce child mortality.

Given this information, and acknowledging that health systems in many countries where IMCI would be most beneficial are weak, almost all IMCI focal persons/NPOs suggested that the approach to IMCI training should be reviewed to avoid nominal implementation: the combination of a long, costly course to be rolled-out in countries with critical health system constraints such as staff shortages may only cripple the reputation of a strategy that could potentially be one of the key interventions to reducing infant and child mortality rates and meeting the fourth Millennium Development Goal.

This paper does not thus raise new issues about challenges to IMCI scale-up; in fact the follow-up recommendations echo the 1998 Follow-Up Guidelines in the IMCI information pack [1], signifying little change since 1998. However this paper is important for three reasons: Firstly this is the first paper to describe challenges to IMCI scale up based on data from all six WHO regions, providing a more global perspective almost twelve years after IMCI was implemented. Thus the paper serves to benchmark the status of IMCI implementation and challenges to scale-up globally. Secondly this paper describes challenges through the eyes of several cadres of health workers from national level to sub-district level. Thirdly the paper presents the results of quantitative and qualitative data on challenges to IMCI scale-up: the latter corroborates the former and of grave concern is that the qualitative data highlights a sense of despondency that surrounds aspects of IMCI scale-up e.g. follow-up after ICMT. This underscores the urgency needed to overcome barriers to IMCI implementation, especially if IMCI is mooted as a key strategy to accelerate progress towards MDG4.

Several approaches to overcoming barriers to IMCI implementation are currently being tested: Since 2009 IMCI Computerised Adaptation and Training Tool (ICATT) - an innovative software technology to support the adaptation of generic IMCI guidelines at national and sub-national levels - has been tested in several settings including Tanzania, Peru, Indonesia and East Java [19]. The course was tested in a classroom setting in Tanzania and Peru and then as distance learning material in Tanzania, Indonesia and East Java. The distance-based course involves three one-day face to face facilitator-participants interactions and two self-learning period in between. During these self-learning periods tutors keep in weekly contact with participants. Early experiences show that difficulties associated with participants' computers and running the ICATT player should be anticipated; facilitators need to be carefully selected and perhaps need to be comfortable with IMCI materials before the course; course duration was too long (56-58 days) and the first contact session was too demanding on facilitators. ICATT is still undergoing further refinement but could be an innovative way to reduce the burden of training both financially and from a human resource perspective. Furthermore in Tanzania D-Tree International has been working to improve the use of the IMCI protocols through the development of an electronic version of IMCI (eIMCI) for use on cell phones and other mobile devices [20]. The software runs on a PDA or mobile phone and guides health workers step-by-step through the full IMCI assessment, classification and treatment plan. The software was designed for ease of use and the training of clinicians took less than 1 hour in all cases. D-Tree have piloted e-IMCI in rural Tanzania where initial results indicate that clinicians were enthusiastic about e-IMCI and more closely adhere to the IMCI protocol when using e-IMCI than without it. A large scale study is currently underway to validate these initial findings that e-IMCI leads to improved adherence to the IMCI protocols compared to the conventional use of IMCI, and to examine the cost and cost-effectiveness of e-IMCI compared to the conventional paper based IMCI and routine ICMT. Furthermore in South Africa a distance IMCI project is being implemented as a major alternative to ICATT and eIMCI.

## Conclusions

Financial challenges to ICMT scale-up and follow-up after training, and human resource shortages (in number or skill) are critical bottle necks to ICMT scale-up. If IMCI is accepted as one of the key strategies to meet MDG4 then ways to reduce cost of ICMT and to integrate follow-up into routine health system organization and functioning should urgently be identified and supported. Cost-effective ways of implementing ICMT need to be explored, including reviewing the competencies, reviewing training approaches such as shortening duration for specific core IMCI competencies, or enhancing on the job-training or distance learning and using technologies such as computer-based training or mobile phone-based training.

## Abbreviations

IMCI: Integrated Management of Childhood Illness Strategy; ICMT: IMCI Case Management Training; WHO: World Health Organisation; UNICEF: United Nations Children's Fund;

## Competing interests

The authors declare that they have no competing interests.

## Authors' contributions

AEG and LMM conceptualized and designed the survey. LMM (WHO) selected the countries and facilities and distributed the questionnaire through IMCI networks.

AEG gathered the data, analysed and interpreted the data and drafted the manuscript.

AEG and LMM contributed to the manuscript finalization. All authors read and approved the final manuscript.

## Pre-publication history

The pre-publication history for this paper can be accessed here:

http://www.biomedcentral.com/1471-2458/11/503/prepub
